# Insights into the Structural Requirements of 2(S)-Amino-6-Boronohexanoic Acid Derivatives as Arginase I Inhibitors: 3D-QSAR, Docking, and Interaction Fingerprint Studies

**DOI:** 10.3390/ijms19102956

**Published:** 2018-09-28

**Authors:** José Luis Velázquez-Libera, Carlos Navarro-Retamal, Julio Caballero

**Affiliations:** Centro de Bioinformática y Simulación Molecular (CBSM), Universidad de Talca, Talca 3460000, Chile; josevlibera2010@gmail.com (J.L.V.-L.); carlos.navarro87@gmail.com (C.N.-R.)

**Keywords:** arginase inhibitors, QSAR, docking, interaction fingerprints

## Abstract

Human arginase I (hARGI) is an important enzyme involved in the urea cycle; its overexpression has been associated to cardiovascular and cerebrovascular diseases. In the last years, several congeneric sets of hARGI inhibitors have been reported with possible beneficial roles for the cardiovascular system. At the same time, crystallographic data have been reported including hARGI–inhibitor complexes, which can be considered for the design of novel inhibitors. In this work, the structure–activity relationship (SAR) of Cα substituted 2(S)-amino-6-boronohexanoic acid (ABH) derivatives as hARGI inhibitors was studied by using a three-dimensional quantitative structure–activity relationships (3D-QSAR) method. The predictivity of the obtained 3D-QSAR model was demonstrated by using internal and external validation experiments. The best model revealed that the differential hARGI inhibitory activities of the **ABH** derivatives can be described by using steric and electrostatic fields; the local effects of these fields in the activity are presented. In addition, binding modes of the above-mentioned compounds inside the hARGI binding site were obtained by using molecular docking. It was found that **ABH** derivatives adopted the same orientation reported for **ABH** within the hARGI active site, with the substituents at Cα exposed to the solvent with interactions with residues at the entrance of the binding site. The hARGI residues involved in chemical interactions with inhibitors were identified by using an interaction fingerprints (IFPs) analysis.

## 1. Introduction

Human arginase (hARG) is the essential enzyme in the last step of the urea cycle of the human body, catalyzing the hydrolysis of l-arginine into l-ornithine and urea. There are two hARG isoforms, named human arginase I (hARGI) and human arginase II (hARGII), which are distributed in different tissues and have different biological functions, but share a similar structure and enzymatic performance [1]. Despite the vital role of hARGI, its overexpression has been related to many diseases, such as cardiovascular [2] and cerebrovascular [3] diseases, which are among the leading causes of death. In this context, hARGI could be considered a potential target in the treatment of these conditions. A problem was found: inhibition of the activity of arginases in affected tissues could disturb the function of the liver. About this, Pudlo et al. [4] indicated that high concentrations of hARGI in the liver are enough for maintaining the function of this organ, while arginases are inhibited in other tissues. Additionally, studies on human patients confirm that administration of arginase inhibitors is beneficial for the cardiovascular system [5].

The list of drug-like hARGI inhibitors is currently short; only a few families of inhibitors have been tested against the activity of this protein [4]. Noteworthy among them are the α-substituted derivatives of 2-amino-6-boronohexanoic acid (ABH), which were reported a few years ago [6,7,8]. **ABH**, an unnatural amino acid, is a potent inhibitor of arginases; its lateral chain has the same length of l-arginine, and the boron at the end of this chain mimics the central carbon of a guanidine group. Boron acts as a Lewis acid when it forms a tetrahedral complex with the nucleophile (H_2_O or OH^−^) coordinated between manganese (II) ions in the binding site of arginases. Golebiowski et al. [6,7,8] synthesized **ABH** α-substituted derivatives by attaching a chemical group R to the α-carbon of **ABH** which resulted in a set of human arginase inhibitors with differential potency, including compounds that improved the activity of **ABH**. They also presented crystallographic structures of several of those compounds inside hARGI and hARGII binding sites, but they limited their theoretical work to the analysis of experimentally obtained structural data.

Theoretical models could help to explore the structure–activity relationships of biologically active ligands. Low computational cost methodologies such as 3D-QSAR (3D-Quantitative Structure–Activity Relationships) and protein–ligand molecular docking could be useful for processing available data from experiments and providing valuable information about the characteristics of the compounds that influence their differential activities. With this in mind, a computational study was performed here to get a better understanding of the structure–activity relationships of **ABH** derivatives as hARGI inhibitors. 3D-QSAR and molecular docking studies were done to extract the chemical features that improve or worsen **ABH** derivative activities and to predict their orientations inside the hARGI binding site. This analysis provides the basis for rational development of novel potent hARGI inhibitors.

## 2. Results

### 2.1. Analysis of 3D-QSAR Models

**Figure 1:**
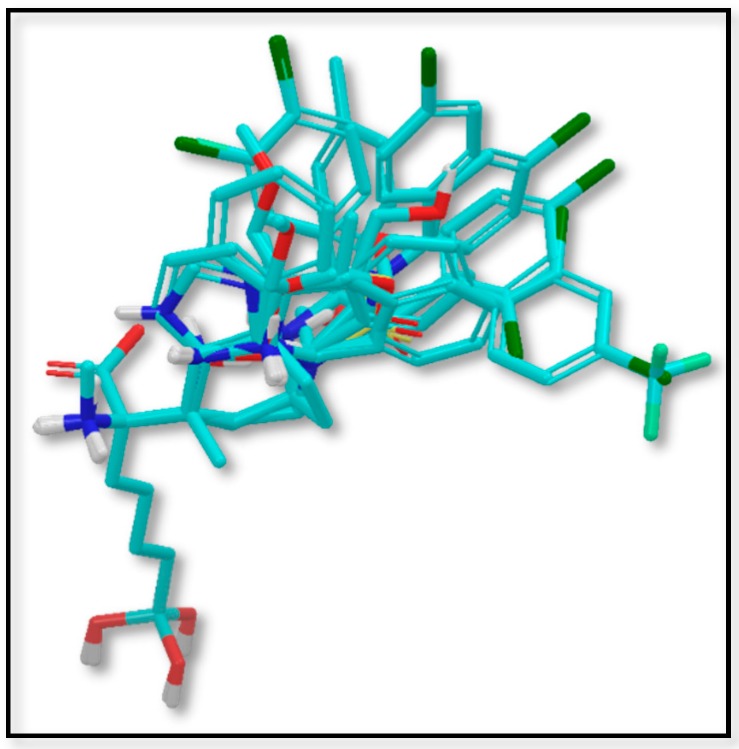
Dataset alignment for 3D-QSAR modeling.

Figure 1 shows the alignment of the 42 molecules (in Table 1) within the grid box used to perform the 3D-QSAR. The alignment shows perfect fitting of the **ABH** region of the molecules and diversity at the region occupied by the substituent at the Cα, which is the region that modulates the structure–activity relationships. It is possible to see that carbon atom replacing the boron of the **ABH** is far from the region with chemical diversity; therefore, no influence of this replacement is expected. By following the above-described 3D-QSAR methodology, three sets of models were computed. Model SE included steric and electrostatic fields; Model S included only the steric field and Model E included only the electrostatic field. In this way, it is possible to evaluate if one field could be irrelevant for modeling, or if both are essential for getting an adequate description of the structure–activity relationships. The statistical quality of the models was evaluated by considering *Q*^2^ values.

**Figure 2:**
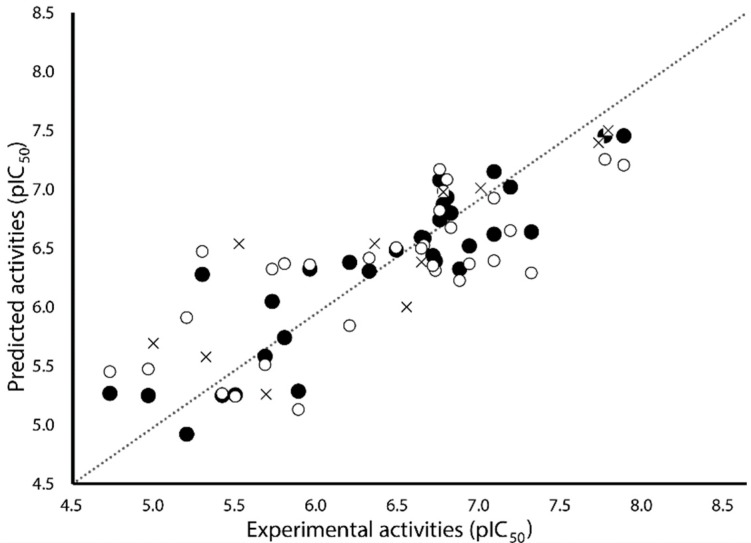
Scatter plot of the experimental activities versus predicted activities for Model SE: (●) training set predictions, (○) LOO-CV predictions, and (×) test set predictions.

Table 2 shows the results for the best 3D-QSAR Models S, E, and SE. As can be appreciated, Model E is not statistically adequate (*Q*^2^ < 0.5). On the other hand, Model S (*Q*^2^ = 0.570, *R*^2^*_test_* = 0.680 and *S_test_ =* 0.487) performed slightly worse than Model SE (*Q*^2^ = 0.572, *R*^2^*_test_* = 0.712 and *S_test_ =* 0.461), mainly in test set predictions. Despite the models S and SE have similar values of *Q*^2^, both steric and electrostatic fields included in Model SE with similar contributions, increased the external predictivity. That is why the ES model, with similar contributions of both fields, was selected as the model best describing the structure–activity relationships of the studied ABH derivatives. Model SE explains 80.2% of the variance and has a low standard deviation (*S* = 0.339). The predictions of pIC_50_ values for the 31 ABH derivatives from the training set using Model SE are reported in Table 1, and the correlations between the predicted and experimental values of pIC_50_ (from training and LOO-CV) are shown in Figure 2. As can be seen, this model fitted well the whole dataset; particularly, the selected model had an outstanding performance when explaining the structure–activity relationships of more potent compounds. The test set predicted pIC_50_ values are listed in Table 1, and the correlations between the predictions and experimental pIC_50_ values are represented in Figure 2. This analysis demonstrated the abilities of Model SE for predicting novel compounds.

**Figure 3:**
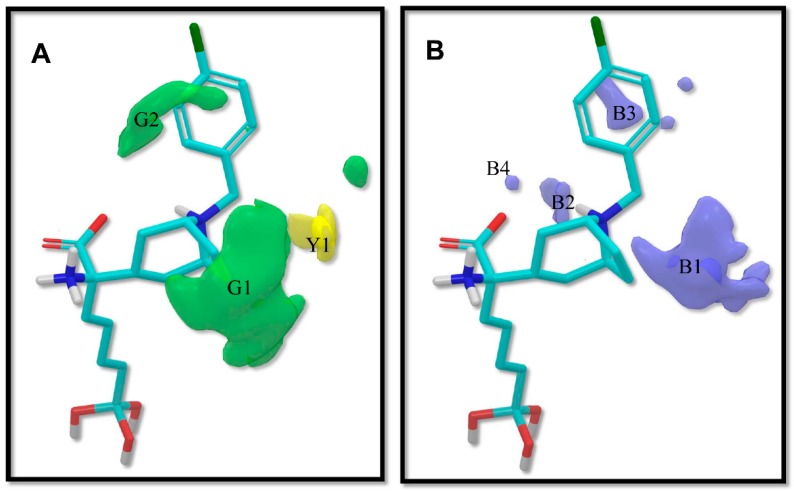
3D-QSAR contour maps for ABH derivatives (SE model): (**A**) steric field, where green isopleths indicate regions where bulky groups enhance the activity, and yellow isopleths indicate regions where bulky groups disfavor the activity; and (**B**) electrostatic field, blue isopleths indicate regions where an increase of positive charge enhances the activity. Compound **p3_11c** is shown inside the fields.

The contour plots of the steric and electrostatic fields are presented in Figure 3 for the modeled hARGI inhibitory activities, where contour plots are represented around the most potent compound **p3_11c** of the dataset. In this figure, green and yellow isopleths indicate regions with positive and negative steric components, respectively, and positive electrostatic terms are represented with blue isopleths (negative electrostatic contours are not visible because they have shallow values).

A green contour (G1 in Figure 3A) near the 8-azabicyclo[3.2.1]octane moiety of compound **p3_11c** indicates that the presence of a bulky group, such as the case of rings and bicycles, near Cα, has a positive effect on the compound’s hARGI inhibitory activity. That is the case of the most potent inhibitors in the dataset, namely **p3_11a**, **p3_11b**, **p3_11c**, **p3_11d**, and **p3_11e** (pIC_50_ between 7.0 and 7.8), which have an 8-azabicyclo[3.2.1]octane group placed in this region. Another large green contour (G2 in Figure 3A) near the 4-Cl-phenyl group of compound **p3_11c** indicates that additional bulky groups at the outer part of the Cα substituents increase hARGI inhibitory activities of the studied compounds. This characteristic is not present in several of the compounds of the series **p2_x**; this series includes the less active compounds in our dataset. In general, compounds with more bulky groups at G2 region are more active; for instance, compound **p1_21** (pIC_50_ = 6.678) contains a methoxymethyl group in this region, while the diasteromer **p1_22** (pIC_50_ = 5.757) contains the same group outside this region. Another example is found when comparing compounds **p1_9** (pIC_50_ = 6.652) and **p1_14** (pIC_50_ = 7.222), which have similar structures, but the last one contains a methyl group at the aminoacidic amine oriented to the G2 region. A unique yellow contour (Y1 in Figure 3A) is located in front of CH_2_ of the benzyl group of compound **p3_11c**, which indicates that bulky groups in this region are not required for increasing hARGI inhibitory activity. For instance, compounds **p1_18** (pIC_50_ = 6.292) and **p1_23** (pIC_50_ = 6.569), which contain tetrahydroisoquinoline and isoindoline groups, respectively, placed in Y1 region, are less active with respect to compound **p1_14** (pIC_50_ = 7.222), which contains the less bulky piperidine group.

On the other hand, blue isopleths are represented in Figure 3B. A blue isopleth (B1 in Figure 3B) near the ethylene of the 8-azabicyclo[3.2.1]octane moiety of compound **p3_11c** indicates that polar groups with positively charge densities have positive contribution to the hARGI inhibitory activity. For instance, compound **p1_27** (pIC_50_ = 7.000), which contains a secondary amine near this region, is one of the most active inhibitors of the series **p1_x**. Compounds **p2_1f** and **p2_1k**, the most active compounds of the series **p2_x**, contain hydrogen bond (HB) donor groups (hydroxyl and primary amine, respectively) which are also near this region. Another blue isopleth (B2 in Figure 3B) near the amine of the 8-azabicyclo[3.2.1]octane moiety of compound **p3_11c** indicates that polar groups in this region with positively charge densities have positive contribution to the hARGI inhibitory activity. That is the case of the most potent inhibitors in the dataset: **p3_11a**, **p3_11b**, **p3_11c**, **p3_11d**, and **p3_11e** (pIC_50_ between 7.0 and 7.8), which have their amine group of 8-azabicyclo[3.2.1]octane ring placed in this region. Another blue isopleth (B3 in Figure 3B) near the 4-Cl-phenyl group of compound **p3_11c** indicates that polar groups with positively charge densities also have positive contribution to the studied activity. For instance, compound **p1_17** (pIC_50_ = 6.638) contains a hydroxyl group near this region having a better activity with respect to compound **p1_16** (pIC_50_ = 5.907) with no group in this region. Finally, a blue isopleth (denoted as B4 in Figure 3B) near the amino acid carboxylate of compound **p3_11c** shows contributions of polar groups in this region to the studied activity. For instance, compounds **p2_1j**, **p2_1k**, and **p2_il**, which are among the most active compounds of the series **p2_x**, contain HB donor NH groups in this region.

### 2.2. Prediction of the Binding Modes

QSAR approximation has many limitations since it only considers the ligands as the structural source, and ignores information of the protein–ligand interactions. That is why, for understanding the SAR in the context of protein binding site, protein–ligand molecular docking methodology could complement the analysis and could give help in guiding the synthesis of new inhibitors. The exploration and analysis of the conformational space of the **ABH** derivatives, restricted by the binding site interactions and taking as reference the experimental data, could lead to more robust protein–ligand interaction models.

**Figure 4:**
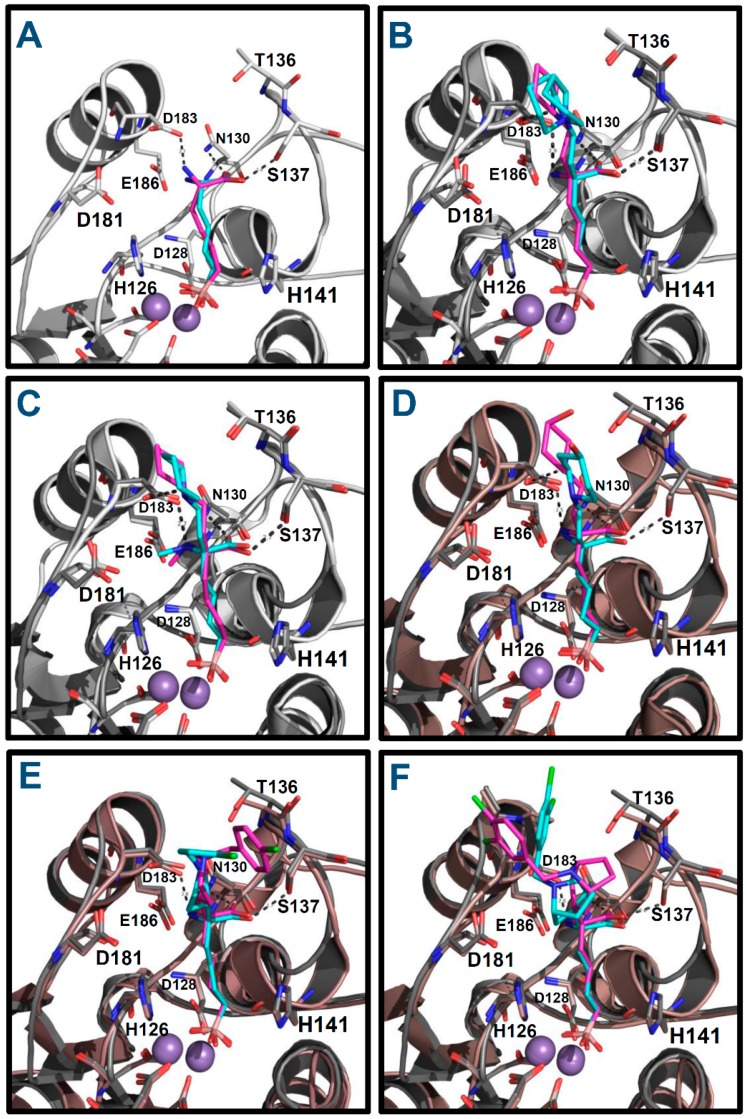
Alignment of docked structures on hARGI–inhibitor X-ray reference structures: (**A**) ABH (reference PDB: 2AEB); (**B**) compound **p1_9** (reference PDB: 4HWW); (**C**) compound **p1_14** (reference PDB: 4HXQ); (**D**) compound **p1_17** (reference PDB: 4IE3, which contains hARGII); (**E**) compound **p3_2d** (reference PDB: 4IXV, which contains hARGII); and (**F**) compound **p3_11d** (reference PDB: 4IXU, which contains hARGII). Crystal structures are represented in cyan; docking results are represented in purple; and hARGI and hARGII structures are represented in gray and brown, respectively.

Firstly, docking poses obtained for **ABH** and compounds **p1_9**, **p1_14**, **p1_17**, **p3_2d**, and **p3_11d** were compared with their conformations in the reference crystallographic structures 2AEB, 4HWW, 4HXQ, 4IE3, 4IXV, and 4IXU, respectively. The experimental conformations in Protein Data Bank (PDB) of **ABH**, **p1_9** and **p1_14** (PDB IDs 2AEB, 4HWW and 4HXQ, respectively) are inside hARGI, and the experimental conformations of **p1_17**, **p3_2d** and **p3_11d** (PDB IDs 4IE3, 4IXV and 4IXU, respectively) are inside hARGII. The structural information in PDB shows that conformations of compounds **p1_9** and **p1_14** crystallized inside hARGI and hARGII have no significant differences [8]; therefore, we consider that our docked poses of **p1_17**, **p3_2d**, and **p3_11d** inside hARGI can be compared with their experimental conformations inside hARGII. Figure 4 shows that the docked structures fitted acceptably with available inhibitor X-ray crystal structures, since all inhibitors with an experimental reference were adequately oriented. The root mean square deviation (RMSD) values for the docked structures with respect to the co-crystal inhibitor structures considering all heavy atoms were <2.0 Å in all these cases (Table 3). If RMSD = 2.0 Å is considered as the threshold value that discriminates between right and wrong docking solutions [9,10], we can state that Glide found suitable binding modes of the ligands in the six cases where a reference is found in PDB.

**Figure 5:**
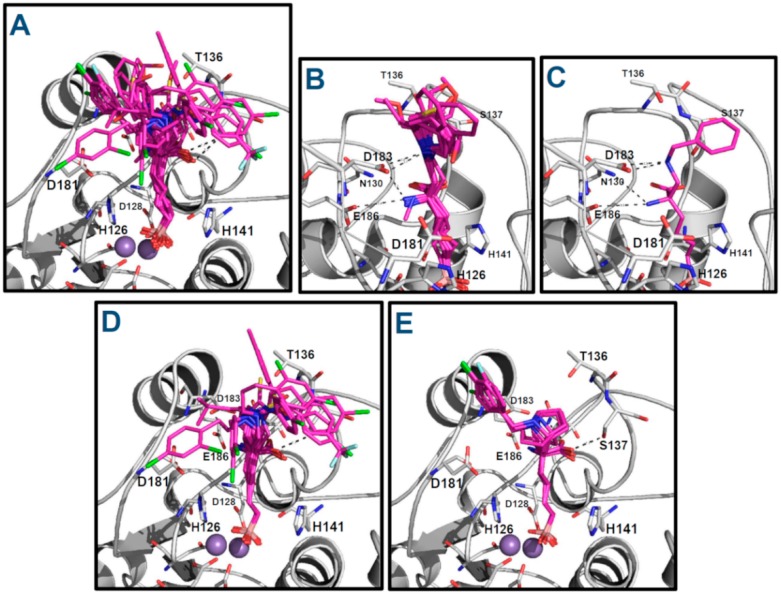
Binding modes of the **ABH** derivatives as hARGI inhibitors: (**A**) binding modes of the whole dataset; (**B**) binding modes of compounds of the series **p1_x** and compound **p2_1m**; (**C**) binding mode of compound **p2_1l**; (**D**) binding modes of compounds of the series **p3_2x**; and (**E**) binding modes of compounds of the series **p3_11x**.

The analysis of the docking of the remaining compounds, following the same protocol, showed similar binding modes (Figure 5); it was expected since all compounds contain the boronate anion group, which is placed in the binding site by mimicking a tetrahedral intermediate of catalysis of arginine hydrolysis. The Glide scoring energy values are reported in Table 1, and they were correlated with experimental pIC_50_ values with *R*^2^ = 0.622.

We also calculated RMSD values for these compounds with respect to the six crystallized references mentioned above by using an *in-house* script. We defined these values as RMSD#PDB, where #PDB refers to the PDB ID of the complex which contains the reference compound. For instance, the bioactive conformation of **p3_11d** inside hARGII is present in PDB with ID 4IXU; therefore, RMSD#PDB values with respect to the conformation of **p3_11d** are named RMSD4IXU in the manuscript. Since **ABH** derivatives, except the own reference (**p3_11d** in the previous example), are different from the reference, RMSD#PDB values were calculated by considering only the common graphs between molecules. In this sense, %RefMatch and %MolMatch values were defined. The %RefMatch values refer to the percent of common graphs between the docked and reference compounds regarding the total number of atoms of the reference compound. The %MolMatch values refer to the percent of common graphs between the docked and reference compounds regarding the total number of atoms of the docked compound. These values allow identifying the maximal similitude between the compared docked and reference compounds; therefore, RMSD#PDB values with high %RefMatch and %MolMatch values indicate that the comparison was established between close structures.

RMSD#PDB values for the studied compounds are reported in Table 4. RMSD2AEB values reflect that the **ABH** group in all compounds had the same orientation (RMSD2AEB < 1.10 Å). The RMSD2AEB %RefMatch values were 100 for all compounds since all of them contain the **ABH** graph. RMSD4HWW values, which define a comparison between the docking poses and the experimental bioactive conformation of compound **p1_9** inside hARGI, are ideal for analyzing the orientations of compounds from series **p1_x** and **p2_x**, because of the higher values of RMSD4HWW %RefMatch and %MolMatch with respect to the values for the other RMSD#PDBs. The common structure between **p1_9** and compounds from the series **p1_x** and **p2_1m** is the *N*-2-aminoethyl-ABH graph; particularly, the common structure between **p1_9** and compounds **p1_14**, **p1_16**, **p1_17**, **p1_18**, and **p2_1m**, is the *N*-1-piperidinylethyl-ABH graph, where the presence of S and O atoms in the six-member ring of **p1_18**, and **p2_1m** is ignored. On the other hand, the common structure between **p1_9** and compounds from the series **p2_x** is the *N*-methyl or *N*-ethyl ABH graphs. Considering these particularities, the RMSD4HWW values are between 0.35 and 1.13 Å for compounds from the series **p1_x** and **p2_1m**. This means that these compounds were oriented similarly to the crystallographic structure of compound **p1_9**, which is the closer reference for calculating an RMSD value for the docking poses of these compounds.

The RMSD4IXV values (Table 4), which define a comparison between the docking poses and the experimental bioactive conformation of compound **p3_2d** inside hARGII, are ideal for analyzing the orientations of compounds from series **p3_2x** because of the higher values of RMSD4IXV %RefMatch and %MolMatch with respect to the values for the other RMSD#PDBs. The common structure between **p3_2d** and compounds from the series **p3_2x** is the *N*-4-piperidinyl-ABH graph. RMSD4IXV values are between 0.63 and 1.55 Å for compounds from the series **p3_2x**. This means that these compounds were oriented similarly to the crystallographic structure of compound **p3_2d**, which is the closest reference for calculating an RMSD value for the docking poses of these compounds.

Finally, RMSD4IXU values (Table 4), which define a comparison between the docking poses and the experimental bioactive conformation of compound **p3_11d** inside hARGII, are ideal for analyzing the orientations of compounds from series **p3_11x**, because the higher values of RMSD4IXU %RefMatch and %MolMatch with respect to the values for the other RMSD#PDBs. The common structure between **p3_11d** and compounds from the series **p3_11x** is the 8-azabicyclo[3.2.1]octane-ABH graph. RMSD4IXU values are between 0.89 and 1.91 Å for compounds from the series **p3_11x**. This means that these compounds were oriented similarly to the crystallographic structure of compound **p3_11d**, which is the closest reference for calculating an RMSD value for the docking poses of these compounds.

The binding orientations of the compounds in Figure 5 show that the best pose for each docked ligand preserved the binding mode of **ABH** within the amino acid recognition region of the hARGI binding site. All of them conserved the **ABH** HB interactions between the ligand’s carboxylate groups and the residues N130 and S137 and between the ligand’s NH_3_^+^ groups and the residue D183. Additionally, all of them have the electrostatic interactions between the ligands B(OH)_3_^−^ groups and Mn^2+^ ions. At the entrance of the binding site, the substituents on Cα are exposed to solvent and could interact freely with water molecules and the protein residues at this region.

A more complete and systematic analysis of the interactions between the docked ligands and hARGI can be performed by using interaction fingerprints (IFPs). They have demonstrated utility in describing the residues involved in forming protein–ligand complexes when they were applied to study other target systems [11]. IFPs are very useful because they capture different types of contacts between a target protein and its ligands. Different chemotypes were defined in IFP calculations such as polar (P), hydrophobic (H), HBs where the residue is acceptor (A), HBs where the residue is donor (D), aromatic (Ar), and electrostatic interactions with charged groups (Ch). The information about contacts with backbone and side-chain functional groups was also provided. First, we calculated IFPs by considering 18 hARGI–inhibitor complexes reported in PDB (PDB IDs 2AEB, 2PLL, 3DJ8, 3F80, 3GMZ, 3GN0, 3KV2, 3LP4, 3LP7, 3MFV, 3SJT, 3SKK, 3THE, 3THJ, 4FCI, 4HWW, 4HXQ, and 4IE1), and then, we performed the same calculation by considering the complexes formed by our 42 docked structures. It is expected that a similitude exists between IFPs for our docking poses and for the hARGI–inhibitor complexes reported in PDB.

**Figure 6:**
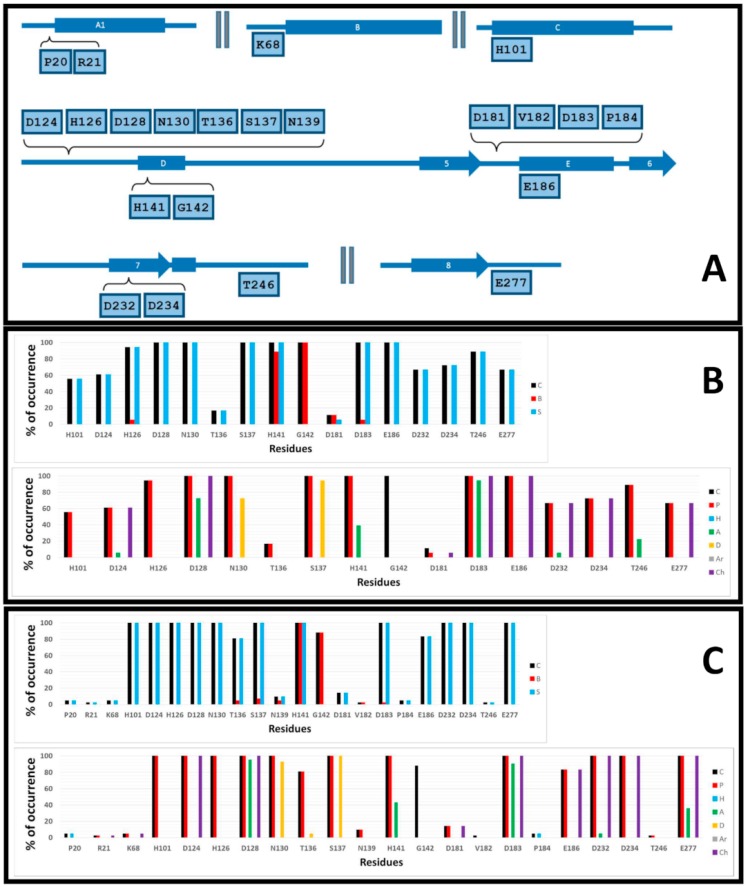
Occurrence of interaction types at the hARGI–ligand binding interface. (**A**) Residues with observed interactions, their position in the hARGI sequence. (**B**) Percent of occurrence of contacts C, interactions with the backbone of the residue B, and interactions with the side chain of the residue S (top); and percent of occurrence of chemical interactions: contacts C, polar P, hydrophobic H, HBs where the residue is acceptor A, HBs where the residue is donor D, aromatic Ar, and electrostatic with charged groups Ch (bottom) for the hARGI–ligand structures reported in PDB. (**C**) The same as (**B**) for the 42 hARGI–ligand structures obtained by docking.

The calculated IFPs are reported in Figure 6. The IFP analysis applied to the 18 hARGI–inhibitor complexes reported in PDB revealed that 16 hARGI residues had contacts with inhibitors (Figure 6B). On the other hand, the IFP analysis applied to the 42 complexes between hARGI and the **ABH** derivatives obtained by docking revealed the contacts of the 16 above-mentioned hARGI residues with inhibitors and the contacts of another six residues with low contributions (Figure 6C). These residues and their positions in hARGI helices and strands are depicted in Figure 6A. The hARGI binding site is very polar; in fact, no hydrophobic or aromatic interactions were observed when analyzing the occurrence of chemical contacts in the structures reported in PDB (Figure 6B).

The residues with interactions with carboxylate and NH_3_^+^ groups of **ABH** were identified in the plots of percent of occurrence obtained from IFP calculations (when we refer to “total structures”, we are considering hARGI–ligand structures reported in PDB and hARGI–ligand structures obtained by docking of **ABH** derivatives inside hARGI binding site):

The residues N130 and S137 at the loop before the helix D have polar contributions in 100% of the total structures. They act as HB donors in more than 70% and 90%, respectively, of the hARGI–ligand structures reported in PDB. percent of HB donor occurrences in our docking complexes were 93% and 100%, respectively.

The residue D183 at the loop before the helix E has polar and electrostatic contributions in 100% of the total structures. It also acts as HB acceptor in more than 90% of the total structures.

Percent of occurrence obtained from IFP calculations for these residues indicated that our docking results conserve the main interactions observed for the available PDB structures: N130 and S137 are HB donors for amino acid carboxylate group of the ligands, and D183 is an HB acceptor for amino acid NH_3_^+^ of the ligands. The following contacts were also observed (Figure 6).

The residues complexing the Mn^2+^ ions have also polar contributions to the bound inhibitors. The residues H101 (at helix C) and H126 (at the loop before helix D) contribute with polar interactions in more than 55% and 100%, respectively, of the PDB structures, and in 100% of the docking complexes. The residues D124, D128, (at the loop before helix D), D232, and D234 (at strand 7) contribute with polar and electrostatic interactions in more than 60% and 100%, respectively, of the PDB structures, and in 100% of the docking complexes. D128 has additional HB acceptor contributions in more than 70% of the PDB structures and in more than 95% of the docking complexes. D124 and D232 also have additional HB acceptor contributions only in 5% of the PDB structures, while D232 have them only in 5% of the docking complexes.

The residue T136 at the loop before helix D has polar interactions in more than 15% of the PDB structures, and in more than 80% of the docking complexes.

The residue H141 at the helix D has polar and HB acceptor interactions in 100% and around 40%, respectively, of the PDB structures and docking complexes. Both side chain and backbone parts of the residue contribute to these interactions.

The residue G142 at the helix D established contacts in 100% of the PDB structures, and in around 90% of the docking complexes.

The residue D181 at the loop before the helix E has electrostatic interactions only in 5% of the PDB structures, and only in around 15% of the docking complexes.

The residue E186 at the helix E has electrostatic interactions in 100% of the PDB structures, and in more than 80% of the docking complexes.

The residue T246 at the loop after the strand 7 has polar interactions in around 90% of the PDB structures, and only in around 2% of the docking complexes. T246 has additional HB acceptor contributions in more than 20% of the PDB structures.

The residue E277 at the loop after the strand 8 has electrostatic interactions in around 65% of the PDB structures and in 100% of the docking complexes. E277 has additional HB acceptor contributions in around 35% of the docking complexes.

About the residue D183, it is important to remark that it not only interacts with the amino NH_3_^+^ group of the inhibitors by a salt bridge, but it is also able to have electrostatic interactions with the protonated tertiary or secondary amines in compounds of the series **p1_x**, compound **p2_1m**, and compound **p2_1l** as can be appreciated in Figure 5B,C. On the other hand, D183 and D181 are essential for supporting the positive charge of the tertiary amine from the piperidine and 8-azabicyclo[3.2.1]octane moiety of compounds of the series **p3_x** (Figure 5D,E).

IFPs identified six additional residues in only a few structures obtained by docking with the following interactions:

The residue P20 at the loop before the helix A1 has hydrophobic contributions in less than 5% of the docked structures, specifically in compounds **p3_2h** (pIC_50_ = 7.222) and **p3_2k** (pIC_50_ = 6.783). 

The residue R21 at the loop before the helix A1 has electrostatic contributions in around 2% of the docked structures (only in compound **p3_2h**).

The residue K68 at the loop before the helix B has electrostatic contributions in less than 5% of the docked structures, specifically in compounds **p3_2j** (pIC_50_ = 6.444) and **p3_2k**.

The residue N139 at the loop before the helix D has polar contributions in around 10% of the docked structures, specifically in compounds **p3_2b** (pIC_50_ = 7.046), **p3_2f** (pIC_50_ = 6.979), **p3_2h**, and **p3_2k**.

The backbone of the residue V182 at the loop before the helix E has contacts in around 2% of the docked structures, only in compound **p3_11d** (pIC_50_ = 7.979).

The residue P184 at the loop before the helix E has hydrophobic contributions in less than 5% of the docked structures, specifically in compounds **p1_22** (pIC_50_ = 5.757) and **p3_11d**.

Noteworthy, the majority of the structures in our study with contacts with these residues are in the group of the most active **ABH** derivatives. It is interesting that NH_3_^+^ side chain group of K68 could have a polar effect on the highly active compounds **p3_2j** and **p3_2k**, which contain a 4-Cl-phenyl group in this region. This finding suggests that replacement of Cl by a negatively charged group could help to increase the binding activity of **p3_2k**. Interestingly, K68 is replaced by valine (V87) in hARGII; therefore, one might think that negatively charged **p3_2k** derivatives could also be hARGI selective inhibitors. However, the residue P20 in hARGI is also replaced by lysine (K38) in hARGII, and this lysine places its NH_3_^+^ side chain group at the same 3D space of the NH_3_^+^ side chain group of K68 from hARGI. Therefore, it is not possible to propose **p3_2k** derivatives with negatively charged groups instead of the 4-Cl-phenyl group as hARGI selective inhibitors.

The IFPs applied to PDB and docked structures demonstrate the reliability of our docking experiments, since the residues identified in the hARGI binding site of the structures determined by X-ray crystallography were also identified in our docking poses. The description of the poses of the **ABH** derivatives studied here, compounds that are recognized as potent hARGI inhibitors could be useful for the design of novel successful inhibitors.

## 3. Materials and Methods

### 3.1. Dataset Collection

Table 1 contains structural representations of the selected compounds for this study (the list of compounds as SMILEs can be found in the Supplementary Materials). Each compound has a unique name, which is formed by the identification for the paper where it was reported, followed by the identification given for the compound in the paper. The dataset was collected from three series of **ABH** derivatives with their hARGI inhibitory values. The same research laboratory group reported the IC_50_ activities against recombinant hARGI in References [8] (compounds named as **p1_x**), [6] (compounds named as **p2_x**), and [7] (compounds named as **p3_x**). The structures were downloaded from the PubChem database [12] and then curated following the procedure recommended by Tropsha in Reference [13]. First, we inspected chemical structures using MarvinView (Marvin 17.1.2.0, 2017, ChemAxon, http://www.chemaxon.com), by matching the downloaded structures with compounds in the above-mentioned literature sources. The resultant dataset of 42 compounds was then processed using Standardizer (JChem 17.1.2.0, 2017, ChemAxon, http://www.chemaxon.com). The protonation states of ionizable groups were calculated and stated at physiological pH using *cxcalc* command line tool, which is implemented in JChem.

### 3.2. QSAR Modeling

Prior to 3D-QSAR models’ elaboration, molecules were aligned by hand in Maestro’s molecular editor (Maestro 10.2.011, Schrödinger LLC, New York, NY, USA), and their IC_50_ values (in M) were converted into logarithmic values log(1/IC_50_) = pIC_50_. For compounds forming racemic mixtures, only R enantiomers were considered, with the exception of compounds **p2_1b** and **p2_1c** (S enantiomers), since their Cα substituents do not differentiate the chiral center configuration with respect to **ABH**. This assumption is plausible taking in account the stereospecificity of arginases for l-enantiomers [4], supported by the reported activity of compound **p1_15** (S-enantiomer with IC_50_ > 300 µM versus IC_50_ = 223 nM for compound **p1_9**, which is the R(l)-enantiomer) in Reference [8].

3D-QSAR models are the result of correlating ligands structural aspects with biological activities, pointing to molecular patterns that could affect the activity in positive and negative ways. The 42 compounds dataset was partitioned into training (31 compounds) and external (11 compounds) sets by the random selection over the space of biological activities which granted the homogenous distribution of selected compounds activities in such space. 3D-QSAR models were generated using Open3DQSAR [14], a versatile 3D-QSAR tool with capacities to process variables, to generate and validate models, and to output and visualize the results. As independent variables, steric and electrostatic fields were computed according to classical molecular mechanics equations using the Merck Molecular Force Field [15], which is implemented in Open3DQSAR [14].

The absence of force field parameters for boron in Merck molecular force field is an important issue for our QSAR modeling. To solve this problem, boron atoms were replaced by carbon atoms in all structures (only for QSAR calculations), in a way similar to the report of Bandyopadhyaya et al. [16]. Fortunately, boron is part of the common region for all the structures and is five bonds from the diverse region (the substituent at the Cα); therefore, this atom is aligned in the same position for all molecules.

The calculated independent variables correspond with the interaction energies between probe atoms (sp_3_ carbon atoms with a charge +1) and structures in a 1.0 Å step size grid box surrounding the whole molecules set. We established the energy cutoffs within 30 kcal/mol and energy values very close to zero (|E| ≤ 0.05 kcal/mol) were set to zero to reduce noise; variables which only assumed a few different values (n-level variables) were also removed. After filtering steps, variables were scaled using the Block Unscaled Weighting procedure [17,18]. Smart Region Definition algorithm was applied to guarantee an improvement of the predictive power of the models [19]. This algorithm groups descriptors into regions of neighbor variables sharing the same chemical and statistical information, removing those regions which do not contribute to increase the predictive power of the models.

*Partial Least Square regression was used to construct 3D-QSAR models, including from one to* five Principal Components and different combinations of fields. For validating the models, we computed the LOO cross-validation procedure, followed by predictions over external validation set (compounds which were not included in modeling).

It should be noted that a template-based alignment 3D-QSAR protocol was employed here according to definition in Reference [20]. These types of protocols have been shown to yield better predictions than those derived from binding site constraint alignments [21], as can be seen in previous works [22,23,24].

### 3.3. Molecular Docking

It is known that **ABH** and its derivatives form a covalent bond with an OH^−^ group to establish electrostatic interactions with Mn^2+^ ions in the binding site [25]. This covalent bond is found in the crystallographic structures used as references in our study (2AEB, 4HWW, 4HXQ, 4IE3, 4IXV, and 4IXU). In this regard, the structures in Table 1 were transformed for docking experiments: their boronic acid groups were changed by tetrahedral boronate anions.

For obtaining the binding modes, ligand–receptor docking calculations were performed using Glide software from Schrödinger suite [26], which has been previously demonstrated good performance in docking of arginase inhibitors [27]. The coordinates of hARGI in the PDB structure with ID 2AEB were used (the complex of hARGI with **ABH**, solved at 1.29 Å of resolution) for constructing the receptor model. This structure′s binding site was compared with the binding sites of the other hARGI records reported in PDB forming complexes with **ABH** derivative inhibitors (PDB IDs: 4HWW, 4HXQ, and 4IE1), without finding any significant difference in the positions of the residues around this cavity. 

Protein structure was prepared using the Protein Preparation Wizard tool implemented in Maestro software of the Schrodinger suite (Protein Preparation Wizard, Schrödinger LLC, New York, NY, USA). Such preparation includes bond order assignments, hydrogen atoms additions, and protonation states predictions of the polar residues. The two Mn^2+^ ions, which are essential for protein–ligand binding, were kept. Then, the system was subjected to molecular minimization using the Impact refinement module [28] and OPLS3 force field [29] with heavy atoms restrained. A grid box of 30 Å × 30 Å × 30 Å was centered on the center of mass of **ABH** to cover the whole binding site (including the residues and consensus water molecules near the Mn^2+^ ions). Glide standard (SP) and extra-precision (XP) modes were used following the same parameters as those used in our previous investigations [30,31,32]. From the found poses, the ones that showed the lower scoring energy and comply with essential chemical interactions described for analogue ligands (ECIDALs) [9,33] for arginase inhibitors were selected as the best poses (one per compound).

IFPs were calculated in the “Interaction Fingerprints Panel” of Maestro (Maestro 10.2.011, Schrödinger LLC, New York, NY, USA) by applying the methodology reported by Singh et al. [34,35]. The presence of different types of chemical interactions between ligands and the binding site residues of the target receptor is accounted by using bits. The binding site for this purpose is defined by distance cutoffs and the interacting set is composed by the residues that have atoms within the specified cutoff distance from ligand atoms. The bits are represented in an interaction matrix, which charts the defined chemical interactions between each ligand and each interacting residue in the receptor.

## 4. Conclusions

Structure–activity relationships and binding orientations of **ABH** derivatives as hARGI inhibitors were studied using 3D-QSAR and molecular docking methods. The 3D-QSAR models were constructed on template aligned molecules, where the **ABH** part of the molecules was completely superposed with the **ABH** compound; therefore, differences identified by 3D-QSAR steric and electrostatic fields were mainly the consequence of the difference in substituents at Cα of **ABH**. Model SE, including both steric and electrostatic fields, had adequate statistical significance, acceptable internal validation statistics (*Q*^2^ = 0.572), and the ability for predicting the compounds left outside the training set (*R*^2^ for the external set 0.712).

On the other hand, docking of the **ABH** derivatives inside hARGI binding site reproduced adequately structural features found in the complexes reported in PDB. All compounds displayed the same orientation and interactions within the active site, where the electrostatic interactions between the ligands B(OH)_3_^−^ groups and Mn^2+^ ions, the HB interactions between the ligands carboxylate groups and the residues N130 and S137, and between the ligands NH_3_^+^ groups and the residue D183 were conserved. Additionally, the substituents on Cα, which explain the differential activities, are exposed to solvent and interact with protein residues in this region. From docking results, a complete map of the hARGI residues which interact with **ABH** derivatives was determined by using IFPs. The information provided here, through the 3D-QSAR and the docking experiments, is useful for increasing the knowledge about **ABH** derivatives and their biological role as hARGI inhibitors, and for improving future proposals.

## Figures and Tables

**Table 1:** Structures of **ABH** analogs as hARGI inhibitors. Experimental and predicted pIC_50_ values using Model SE and docking Glide scoring energy values.

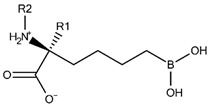					
Compound	R1	R2	Experimental pIC_50_ (hARGI)	Predicted pIC_50_ (hARGI)	Scoring Energies (kcal/mol)
**ABH**	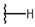	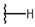	5.839	5.286	−7.519
**p1_9**	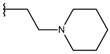	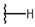	6.652	6.390	−5.375
**p1_14**	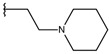	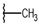	7.222	6.639	−7.654
**p1_16**	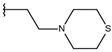	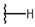	5.907	6.324	−5.617
**p1_17**	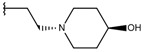	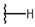	6.638	6.436	−4.563
**p1_18** ^1^	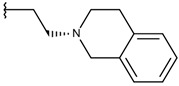	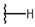	6.292	6.538	−5.527
**p1_19**	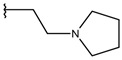	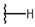	6.796	6.323	−6.365
**p1_20**	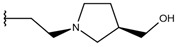	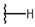	6.585	6.585	−5.238
**p1_21**	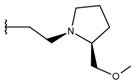	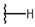	6.678	6.742	−4.378
**p1_22**	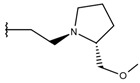	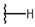	5.757	5.741	−5.582
**p1_23**	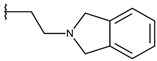	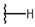	6.569	6.591	−5.649
**p1_24**	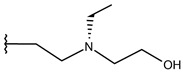	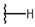	6.260	6.306	−4.098
**p1_25** ^1^	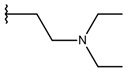	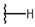	6.569	6.382	−5.631
**p1_26**	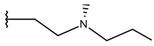	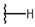	6.854	6.520	−5.731
**p1_27**	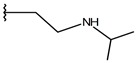	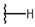	7.000	6.619	−5.697
**p2_1b**	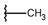	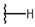	5.465	5.254	−2.985
**p2_1c**	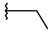	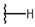	5.387	5.251	−3.064
**p2_1d**	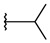	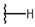	4.949	5.249	−3.258
**p2_1e** ^1^	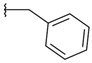	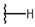	4.979	5.693	−3.605
**p2_1f** ^1^	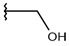	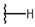	5.648	5.260	−2.868
**p2_1g**	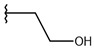	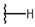	4.721	5.268	−2.682
**p2_1i** ^1^	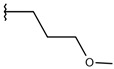	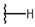	5.291	5.578	−2.791
**p2_1j**	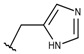	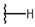	5.642	5.583	−3.166
**p2_1k**	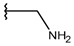	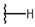	5.684	6.048	−2.993
**p2_1l** ^1^	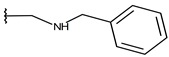	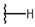	5.487	6.538	−5.414
**p2_1m**	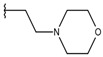	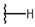	5.269	6.277	−4.513
**p3_2a**	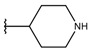	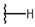	6.420	6.485	−8.167
**p3_2b**	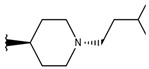	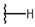	6.745	6.799	−6.672
**p3_2c**	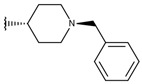	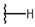	6.699	6.873	−7.551
**p3_2d**	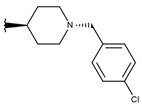	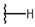	6.721	6.932	−7.622
**p3_2e** ^1^	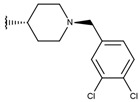	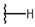	6.699	6.977	−6.540
**p3_2f**	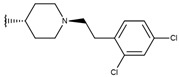	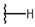	6.678	7.079	−6.116
**p3_2g**	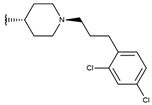	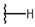	7.000	7.150	−6.206
**p3_2h** ^1^	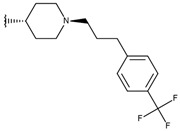	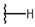	6.921	7.011	−6.763
**p3_2i**	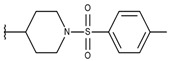	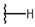	5.177	4.921	−4.324
**p3_2j**	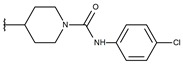	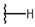	6.143	6.380	−5.062
**p3_2k** ^1^	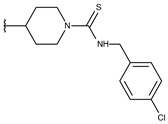	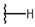	6.481	6.001	−4.897
**p3_11a**	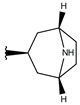	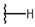	7.097	7.020	−8.395
**p3_11b** ^1^	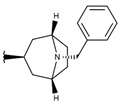	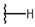	7.620	7.396	−8.075
**p3_11c**	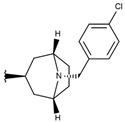	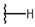	7.770	7.455	−8.060
**p3_11d** ^1^	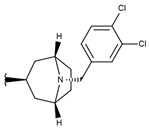	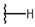	7.678	7.500	−7.980
**p3_11e**	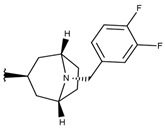	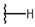	7.658	7.456	−6.961

^1^ Test set compounds.

**Table 2:** 3D-QSAR analysis results.

Model	*NC*	*R* ^2^	*S*	*Q* ^2^	*S_CV_*	Fraction	
						Steric	Electrostatic
S	5	0.860	0.285	0.570	0.497	1	0
E	3	0.784	0.354	0.464	0.557	0	1
SE	3	0.802	0.339	0.572	0.497	0.460	0.540

*NC* is the number of components from the PLS analysis; *R*^2^ is the square of the correlation coefficient; *S* is the standard deviation of the regression; and *Q*^2^ and *S*_cv_ are the correlation coefficient and standard deviation, respectively, of the leave-one-out (LOO) cross-validation.

**Table 3:** Docking accuracy of compounds with a reference in PDB.

PDB Code of the Complex	Co-Crystallized Inhibitor	Co-Crystallized Enzyme	RMSD Value (Å)
2AEB	**ABH**	hARGI	1.07
4HWW	**p1_9**	hARGI	1.09
4HXQ	**p1_14**	hARGI	1.04
4IE3	**p1_17**	hARGII	1.59
4IXV	**p3_2d**	hARGII	1.55
4IXU	**p3_11d**	hARGII	1.91

**Table 4:** RMSD#PDB values of the obtained docking pose common fragments for the studied compounds with respect to available co-crystallized hARGI inhibitors (identified by their #PDB codes) as references.

	ABH as Reference in 2AEB			p1_9 as Reference in 4HWW			p1_14 as Reference in 4HXQ			p1_17 as Reference in 4IE3			p3_2d as Reference in 4IXV			p3_11d as Reference in 4IXU		
Compound	RMSD2AEB ^1^ (Å)	%RefMatch ^2^	%MolMatch ^3^	RMSD4HWW ^1^ (Å)	%RefMatch ^2^	%MolMatch ^3^	RMSD4HXQ ^1^ (Å)	%RefMatch ^2^	%MolMatch ^3^	RMSD4IE3 ^1^ (Å)	%RefMatch ^2^	%MolMatch ^3^	RMSD4IXV ^1^ (Å)	%RefMatch ^2^	%MolMatch ^3^	RMSD4IXU ^1^ (Å)	%RefMatch ^2^	%MolMatch ^3^
**ABH**	**1.07 ^4^**	**100**	**100**	1.06	62	100	1.05	59	100	1.13	59	100	1.08	48	100	1.08	43	100
**p1_9**	0.89	100	62	**1.09 ^4^**	**100**	**100**	1.02	95	100	1.53	95	100	0.91	52	67	0.87	47	67
**p1_14**	0.92	100	59	1.11	100	95	**1.04 ^4^**	**100**	**100**	1.55	95	95	0.88	52	64	0.94	47	64
**p1_16**	0.89	100	62	1.13 ^5^	100	100	1.04 ^5^	95	100	1.56 ^5^	95	100	0.90	52	67	0.87	47	67
**p1_17**	0.91	100	59	1.07	100	95	0.99	95	95	**1.59 ^4^**	**100**	**100**	0.94	52	64	0.90	47	64
**p1_18**	0.89	100	52	1.01	100	84	0.96	95	84	1.46	95	84	0.91	52	56	0.87	47	56
**p1_19**	0.95	100	65	0.93	76	80	0.96	73	80	1.13	73	80	0.95	52	70	0.93	47	70
**p1_20**	0.89	100	59	0.85	76	73	0.85	73	73	1.10	73	73	0.91	52	64	0.87	47	64
**p1_21**	0.30	100	57	0.41	76	70	0.44	73	70	0.81	73	70	0.47	52	61	0.35	47	61
**p1_22**	0.95	100	57	0.89	76	70	0.92	73	70	1.07	73	70	0.94	52	61	0.93	47	61
**p1_23**	0.89	100	54	0.88	76	67	0.90	73	67	1.11	73	67	0.92	52	58	0.87	47	58
**p1_24**	0.37	100	62	0.44	76	76	0.44	73	76	0.87	73	76	0.51	52	67	0.41	47	67
**p1_25**	0.25	100	65	0.37	76	80	0.38	73	80	0.80	73	80	0.40	52	70	0.28	47	70
**p1_26**	0.32	100	65	0.46	76	80	0.48	73	80	0.86	73	80	0.44	52	70	0.34	47	70
**p1_27**	0.23	100	68	0.35	76	84	0.37	73	84	0.78	73	84	0.37	52	74	0.27	47	74
**p2_1b**	0.98	100	93	1.00	67	100	0.98	64	100	1.05	64	100	0.98	52	100	1.06	47	100
**p2_1c**	0.98	100	87	0.99	71	100	0.96	68	100	1.06	68	100	0.97	52	93	1.04	47	93
**p2_1d**	0.92	100	81	0.87	71	94	0.88	68	94	1.13	68	94	0.94	52	88	0.93	47	88
**p2_1e**	0.88	100	65	0.92	71	75	0.94	68	75	1.06	68	75	0.91	52	70	0.88	47	70
**p2_1f**	0.96	100	87	0.98	67	93	0.97	64	93	1.03	64	93	0.96	52	93	1.03	47	93
**p2_1g**	0.89	100	81	0.85	71	94	0.85	68	94	1.07	68	94	0.91	52	88	0.88	47	88
**p2_1i**	0.24	100	72	0.27	71	83	0.31	68	83	0.69	68	83	0.25	52	78	0.34	47	78
**p2_1j**	0.92	100	68	0.91	71	79	0.89	68	79	1.12	68	79	0.90	52	74	1.00	47	74
**p2_1k**	0.32	100	87	0.36	67	93	0.38	64	93	0.70	64	93	0.44	52	93	0.34	47	93
**p2_1l**	0.35	100	59	0.39	67	64	0.48	64	64	0.64	64	64	0.40	52	64	0.37	47	64
**p2_1m**	0.55	100	62	1.03 ^5^	100	100	0.95 ^5^	95	100	1.53 ^5^	95	100	0.63	52	67	0.56	47	67
**p3_2a**	0.87	100	68	0.86	67	74	0.87	64	74	1.06	64	74	0.87	70	100	1.20	63	100
**p3_2b**	0.98	100	54	1.01	67	58	0.97	64	58	1.23	64	58	1.11	78	88	1.60	70	88
**p3_2c**	0.19	100	50	0.22	67	54	0.28	64	54	0.67	64	54	1.41	96	100	3.36	87	100
**p3_2d**	0.88	100	48	0.86	67	52	0.86	64	52	1.06	64	52	**1.55 ^4^**	**100**	**100**	3.86	90	100
**p3_2e**	0.89	100	46	0.89	67	50	0.88	64	50	1.10	64	50	1.44	100	96	4.22	93	100
**p3_2f**	1.02	100	45	1.03	67	48	1.01	64	48	1.22	64	48	1.38	78	72	1.36	70	72
**p3_2g**	0.49	100	43	0.57	67	47	0.65	64	47	0.36	64	47	1.29	78	70	1.91	70	70
**p3_2h**	0.88	100	41	0.87	67	44	0.89	64	44	1.05	64	44	1.24	78	66	1.81	70	66
**p3_2i**	0.32	100	45	0.31	67	48	0.32	64	48	0.73	64	48	0.63	70	66	1.11	63	66
**p3_2j**	0.91	100	45	0.88	67	48	0.87	64	48	1.10	64	48	1.15	74	69	1.70	67	69
**p3_2k**	0.26	100	43	0.30	67	47	0.31	64	47	0.71	64	47	0.68	74	67	1.31	67	67
**p3_11a**	0.24	100	62	0.23	67	67	0.31	64	67	0.69	64	67	0.87	70	90	0.89	70	100
**p3_11b**	0.27	100	46	0.28	67	50	0.36	64	50	0.62	64	50	2.94	96	93	1.51	93	100
**p3_11c**	0.91	100	45	0.87	67	48	0.85	64	48	1.12	64	48	3.79	100	93	1.88	97	100
**p3_11d**	0.27	100	43	0.25	67	47	0.23	64	47	0.71	64	47	3.66	100	90	**1.91 ^4^**	**100**	**100**
**p3_11e**	0.29	100	43	0.31	67	47	0.41	64	47	0.61	64	47	2.80	96	87	1.32	93	93

^1^ RMSD values considering only the common chemical fragments between the docked compound and the reference compound. ^2^ %RefMatch refers to the percent of common graphs between the docked and reference compounds concerning the total number of atoms of the reference compound. ^3^ %MolMatch refers to the percent of common graphs between the docked and reference compounds regarding the total number of atoms of the docked compound. ^4^ This RMSD#PDB value represents the classical RMSD value between an obtained docking pose and the same compound in a crystallographic PDB structure; it happens when both %RefMatch and %MolMatch are 100. ^5^ In this case, difference in ring heavy atoms were not considered between the docked compound and the reference compound.

## Supplementary Materials

Supplementary materials can be found at http://www.mdpi.com/1422-0067/19/10/2956/s1.

## Abbreviations

3D	three-dimensional
ABH	2(S)-amino-6-boronohexanoic acid
hARGI	human arginase I
IFP	interaction fingerprints
LOO	leave-one-out
PDB	Protein Data Bank
RMSD	root mean square deviation
SAR	structure–activity relationship
